# Does fear-of-failure mediate the relationship between educational expectations and stress-related complaints among Swedish adolescents? A structural equation modelling approach

**DOI:** 10.1093/eurpub/ckad200

**Published:** 2023-11-15

**Authors:** Matthew R Cashman, Mattias Strandh, Björn Högberg

**Affiliations:** Department of Social Work, Umeå University, Universitetstorget 4, Umeå 901 87, Sweden

## Abstract

**Background:**

This study investigated the possible mediating role of fear-of-failure between educational expectations and adolescent stress-related complaints with a specific focus on gender differences among Swedish adolescents, and related these findings more broadly to school-related demands and stress-related complaints.

**Methods:**

A total of *N* = 5504 Swedish adolescents (*M*_age_* = *15 years, SD = 0.0 years, 50.2% girls) were drawn from the 2018 Swedish Programme for International Student Assessment study for our investigation. We used structural equation models to explore if fear-of-failure mediates the relationship between educational expectations and negative affect, with a specific focus on gender differences. Educational expectations were utilized in the measurement model. Fear-of-failure was constructed as a latent mediating variable. Negative affect was constructed as a latent variable and utilized as an outcome variable. We subsequently undertook bootstrapping tests of indirect effects and non-linear comparisons of indirect effects to assess the reliability of the results.

**Results:**

Fear-of-failure partially mediated the association between educational expectations and negative affect (39%). Our gender-specific structural equation model demonstrated that this relationship was more pronounced for girls, suggesting girls are more vulnerable to negative affect as a result of experiencing higher levels of fear of failing.

**Conclusions:**

The findings suggest that fear-of-failure partially explains the association between educational expectations and negative affect and that this association is more pronounced for girls. This study provides insights into better understanding adolescent stress-related complaints, and the differential role fear of failing has in regards to gender.

## Introduction

Mental health complaints are common among adolescents and are typically stress-related or induced.^1^ Stress-related complaints arise when the demands experienced by individuals exceed their perceived ability to manage these demands.^2^^,^^3^ Rates of academic stress have increased significantly in high-income countries, including Sweden, the focus of this study.^4^ Experiences of stress-related complaints affect adolescent girls to a greater degree than boys, with the Swedish Public Health Agency contending that 24% of girls report frequent stress-related symptoms compared with 11% of boys.^5^ Recent research suggests that perceived school demands largely explain documented differences in girls’ stress levels compared with boys, as girls fear living up to academic demands more than boys.^2^ This in turn is largely explained by the high educational aspirations of girls compared with boys.^6^ Thus, girls likely increase academic effort, causing achievement to be high, while simultaneously generating increased stress.^7–9^ Experiences of stress likely impair day-to-day functioning and may result in persistent mental health issues in adulthood.^1^

Considering the adverse consequences of stress on adolescent health, it is important to investigate other important underlying factors that may increase stress levels, and gender inequalities in stress among adolescents. Recent findings from the Programme for International Student Assessment Survey (PISA) found that over half of all students in education systems surveyed expressed fear-of-failure, and that this is more pronounced for girls.^10^ Fear-of-failure is determined by the perceived risk of failing and the subsequent costs associated with failure.^11^ Fear-of-failure has been found to be negatively associated with lower social and emotional well-being among students, including higher levels of stress.^11^ Stress-related symptoms among students are strongly related to a lack of perceived achievement and increasing educational expectations, especially among girls.^12^ Further, higher levels of fear of failing are associated with lower levels of life satisfaction and poorer concentration^10^ and may therefore have adverse effects on both health and academic outcomes.

Previous research aiming to investigate the role fear-of-failure may have on mental health-related outcomes in Sweden is limited. International findings suggest that girls tend to report higher levels of fear-of-failure and are more likely to experience stress, anxiety, burnout and depression compared with boys.^11^ Furthermore, despite both boys and girls experiencing fear-of-failure, girls are more likely to experience subsequent fears of shame and embarrassment, which may lead to an increased likelihood of withdrawing academically due to ‘achievement guilt’.^13^ Further, research suggests that fear-of-failure may act as a ‘two-edged sword’, by which experiencing fear-of-failure boosts students’ academic performance, but simultaneously reduces their overall life satisfaction and socio-emotional well-being.^14^ This association was more pronounced among girls, supporting previous findings that girls tend to exert greater academic effort than boys, but in turn experience higher levels of stress and other mental health outcomes due to this greater effort.^7^^,^^15^ Following this research background, this study seeks to extend previous findings in a Swedish context and proposes the following aim and research questions.

### Aims

The aim of this article is to investigate whether fear-of-failure mediates the relationship between educational expectations and negative affect, with a special focus on gender differences. The research questions are as follows:

1. Does fear-of-failure mediate the relationship between educational expectations and negative affect?
2. Does the strength of this mediation differ by gender?

## Methods

Data from the Swedish 2018 PISA survey were used for this study. PISA is a repeated cross-sectional survey that aims to gather cross-country, comparable data on 15-year-old student’s ability to use their reading, mathematics, science knowledge and perceived skills to meet real-life challenges.^10^ PISA is conducted using a random cluster design, where schools are the primary sampling unit. This cluster design comprises the random selection of schools with students aged 15 years, of which 37 students are randomly selected in each school. Controversy has arisen regarding the Swedish 2018 PISA results as the exclusion rate exceeded the 5% threshold. It is estimated that roughly 2.4% of the weighted number of participating students in Sweden were excluded due to issues surrounding immigration and learning disabilities.^16^ Despite technical reviews maintaining Sweden met all technical standards despite these exclusions, it is possible that adolescent immigrants and those with learning disabilities are underrepresented in this data.^16^ In our study, a total of 5504 participants were included in the analysis (*M*_age_* = *15 years, SD* *=* *0.0 years, 50.2% girls) 87.9% were born in Sweden, while the remaining participants migrated to Sweden prior to undertaking upper-secondary school, and had been taught in the assessment language of Swedish for at least a year as required by PISA technical standards.^16^ The socio-economic status of the Swedish sample is slightly higher than the OECD average of 0 (*M*_SES_*=* 0.362, SD = 0.88) as measured by a three-way index of economic, social and cultural status. An expanded descriptive summary table of our sample is provided in table 1.

**Table 1:** Descriptive summary statistics

Variable	*N*	%	Mean	SD
Age: 15	5504	100		
Student grade				
Grade 8	108	1.96		
Grade 9	5334	96.9		
Grade 10	62	1.13		
Sex				
Male	2741	49.8		
Female	2763	50.2		
Life satisfaction	5211		7.00	2.53
Economic and social cultural status	5348		0.362	0.891
Immigration status				
Native	4283	80.2		
Second generation	556	10.4		
First generation	499	9.35		
Plausible reading value	5504		505.4	106.4
Social media use				
Never/hardly never	174	3.63		
Once/twice a month	156	3.26		
Once/twice a week	274	5.72		
Almost everyday	671	14.01		
Everyday	3513	73.3		
Parental emotional support	4924		−0.11	0.972

### Measures

#### Educational expectations

We measured students’ educational expectations utilizing a single indicator questioning what level of education individuals expect to complete: ‘<ISCED level 5A or 6>’, which corresponds to a bachelor’s degree or equivalent internationally.

#### Negative affect

PISA does not contain a specific indicator measuring stress. Therefore, we utilized PISA’s students’ feelings battery which measures negative affect as a proxy for stress-related complaints: ‘Thinking about yourself and how you normally feel: how often do you feel as described below?’, ‘scared’, ‘miserable’, ‘afraid’ and ‘sad’, with available responses ranging from ‘never’ (1) to ‘always’ (4). Previous research has demonstrated that stress and negative affect are closely associated and share similar measurement consistency in regard to negative emotional states.^17^ Moreover, in qualitative studies, adolescents themselves describe stress broadly as referring to negative psychological and emotional states.^17^ The consistency of the latent variable was tested with confirmatory factor analysis (CFA). Factor loadings ranged from 0.63 to 0.77, which demonstrates the indicators extract sufficient variance in measuring negative affect or stress-related complaints.

#### Fear-of-failure

Fear-of-failure was constructed from three indicators: ‘When I am failing, I worry what others think of me’, ‘When I am failing, I am afraid that I might not have enough talent’ and ‘When I am failing, this makes me doubt my plans for the future’, with available responses ranging from ‘strongly disagree’ (1) to ‘strongly agree’ (4). The consistency of the latent variable was tested with CFA. Factor loadings ranged from 0.72 to 0.93, which demonstrates the indicators extract sufficient variance in measuring fear-of-failure.

### Control variables

We included five control measures to ensure that the observed relationships between our measures were not confounded by other factors. A detailed description of these variables is provided in a Supplementary file S1.

#### Immigration status

We control for immigration as previous studies have demonstrated that ethnic background may have an effect on educational expectations and achievement desires.^10^

#### Index of economic, social and cultural status

We control for the effects of economic, social and cultural status in our structural equation model by utilizing PISA’s three-way index measure.

#### Parental emotional support

We control for the effects of parental emotional support by utilizing PISA’s parental emotional support construct, as previous studies have demonstrated that emotional support from parental figures may affect educational expectations and stress.^10^

#### Reading achievement

We control for adolescents objective PISA reading score to ensure that educational expectations are not being adjusted by individuals’ academic ability.

#### Social-media use

We control for the potential effects social media use may have on students’ well-being. Previous research has indicated that increased use of social media platforms may promote increased levels of anxiety, depression and stress.^18^

### Analytic strategy

Structural equation modelling was performed to investigate our research questions assessing if fear-of-failure mediates the relationship between educational expectations and negative affect. We subsequently undertook a multi-group structural equation model using gender as a grouping variable to test specific gender differences in the strength of this association. All models utilized final student sampling weights in order to calculate unbiased estimates and sampling error inferences among the population and utilized maximum likelihood missing values estimation to account for missing data. Clustered robust standard errors were utilized to adjust for PISA’s clustered data structure on error estimates. Clustered robust standard errors is an accepted method to adjust for overstated error estimates among clustered data and generates similar standard errors when utilizing multi-level models or hierarchical linear models.^19^

## Results

Our results demonstrated that the overall goodness-of-fit for our model meets the required cut-off values for acceptable model fit. Our root mean square error of approximation fell within the recommended range of 0.08 and 0.05, with values closer to 0 representing a good fit. Both the comparative fit index and the Tucker-Lewis Index fell above the accepted value of 0.90. Likelihood ratios were not reported due to the distorting effect of large sample sizes when running structural equation models.^20^

The results suggest that educational expectations are associated with higher levels of negative affect among Swedish adolescents (*B* = 0.03, *P* < 0.005). Educational expectations demonstrated a statistically significant positive relationship with fear-of-failure (*B* = 0.04, *P* < 0.005) suggesting higher educational expectations are associated with increased fears-of-failure. A statistically significant positive relationship was demonstrated between fear-of-failure and negative affect (*B* = 0.47, *P* < 0.001) suggesting higher levels of fear-of-failure are associated with greater levels of negative affect. Our structural equation model demonstrating the standardized coefficients between educational expectations, fear-of-failure, negative affect and our control measures are provided in Supplementary table S2.

In regard to research question 1, we undertook a Baron and Kenny^21^ mediation test of indirect effects to ascertain the mediating role of fear-of-failure on negative affect. We additionally implemented a supplementary bootstrapping significance test recommended by Zhao et al.^22^ to ascertain the accuracy of mediation (table 2). According to the Baron and Kenny test, fear-of-failure partially mediated the relationship between educational expectations and negative affect*.* The ratio of the indirect effect to the total effect (0.020/0.051 = 0.386) demonstrated that 39% of the effect of educational expectations on negative affect is mediated by fear-of-failure. Additionally, when accounting for the ratio of the indirect to the direct effect (0.020/0.047 = 0.628), the mediated effect was roughly 0.6 time as large as the direct effect of educational expectations on negative affect. Furthermore, the bootstrapping significance test^22^ further supported the significance of this mediation, as the 95% confidence interval did not include or overlap with zero (table 2).

**Table 2:** Bootstrapping significance test of indirect effects

	Observed coefficient	Bias	Bootstrap SE	95% Confidence interval		
Indirect effect	0.02981377	0.0004243	0.00740187	0.0153065	0.044321	Normal
				0.0163231	0.046717	Percentile
				0.0172109	0.049857	Bias-corrected

In regard to research question 2, we undertook a gender-specific multi-group structural equation model to investigate if the mediated effect differs between boys and girls, presented in table 3. Our results indicated that girls experience greater levels of fear-of-failure (*B* = 0.18, *P* < 0.001) when experiencing high levels of educational expectations compared with boys (*B* = −0.09, *P* < 0.001). Indeed, boys experienced reduced levels of fear-of-failure when experiencing educational expectations, while also experiencing reduced levels of negative affect (*B* = −0.11, *P* < 0.001). Girls demonstrated increased levels of negative affect when experiencing higher educational expectations (*B* = 0.11, *P* < 0.001). However, both boys (*B* = 0.43, *P* < 0.001) and girls (*B* = 0.51, *P* < 0.001) experienced increased levels of negative affect when experiencing greater levels of fear-of-failure.

**Table 3:** Gender-specific multi-group structural equation model estimates

	Observed coefficient	Bootstrap SE	*z*	*P* > |*z*|	95% Confidence interval	
Boys (*N* = 2664)						
FoF ← Edu-Exp	−0.0881706	0.0160432	−5.50	0.000	−0.1196147	−0.0567265
Neg-A ← FoF	0.434095	0.0248246	17.49	0.000	0.3854397	0.4827504
Neg-A ← Edu-Exp	−0.1109197	0.0148922	−7.45	0.000	−0.1401078	−0.0817315
Girls (*N* = 2703)						
FoF ← Edu-Exp	0.1756897	0.0164091	10.71	0.000	0.1435285	0.2078509
Neg-A ← FoF	0.5091498	0.0246294	20.67	0.000	0.4608772	0.5574225
Neg-A ← Edu-Exp	0.1070438	0.0149562	7.16	0.000	0.0777303	0.1363574

Notes: Edu-Exp, educational expectations; FoF, fear-of-failure; Neg-A, negative affect.

In order to assess if the mediating role of fear-of-failure differs between boys and girls, we undertook a non-linear comparison test of indirect effects (table 4). This test demonstrates that the difference in this mediation is statistically significant as the 95% confidence interval does not include zero.

**Table 4:** Non-linear comparison of indirect effects

	Indirect coefficient	SE	*Z*	*P* > |*z*|	95% Confidence interval	
Boys	−0.0382744	0.0086623	−4.42	0.000	−0.552522	−0.0212966
Girls	0.0894524	0.0094833	9.43	0.000	0.0708654	0.1080393

## Discussion

This study explored if fear-of-failure mediates the relationship between educational expectations and negative affect, with a special focus on gender differences among Swedish adolescents. Research question one sought to answer if fear-of-failure mediates the relationship between educational expectations and negative affect. Results showed that fear-of-failure partially mediates this relationship, as 39% of the effect of educational expectations on negative affect was mediated by fear-of-failure. Research question two sought to answer if the strength of this mediation differed by gender. Results showed that fear-of-failure is more associated with negative affect among girls than boys. Specifically, our results demonstrated that educational expectations were associated with lower levels of fear-of-failure and negative affect among boys. Our findings therefore suggest girls are more vulnerable to experiences of negative affect when possessing higher educational expectations, which appears to increase due to increased levels of fear-of-failure in an educational setting. Our findings support previous studies which suggest that adolescents experiencing fear-of-failure tend to demonstrate poorer mental health outcomes.^23^ Our findings additionally support previous studies which indicate that girls are more likely to experience and report fear-of-failure and subsequently poorer mental well-being following experiences of fear-of-failure.^11^ Importantly, our study supports previous findings which suggest that girls tend to exert greater academic effort when facing increasing demands and expectations, which in turn results in pronounced levels of stress and negative emotional states.^7^^,^^15^

Though this study utilized educational expectations in the empirical models, we consider educational expectations to be a dimension of demands more generally. Individuals expecting or aspiring to achieve this level of education can in turn be interpreted as possessing higher levels of school-related demands in order to meet these expectations. As such, higher educational expectations are likely a dimension of demands more generally. This conceptualization is supported by recent investigations which demonstrate that individuals possessing higher personal academic expectations experience greater levels of perceived demands, as educational success is viewed as the primary resource to secure one’s desired future^24^ as perceived school-related demands are likely caused by possessing high educational expectations.^24^ Similarly, we consider negative affect to encompass stress-related complaints more generally, as both stress-related complaints and negative affect share similar measurement consistency in regard to negative emotional states.^17^ Furthermore, adolescents themselves generally refer to stress as reflecting unpleasant psychological and negative emotional states.^17^ As a result, we discuss our findings in relation to school-related demands and stress-related complaints more broadly and associate these findings with this literature base.

Swedish adolescent’s higher levels of stress-related complaints due to increased fears-of-failure are likely explained by multiple interrelated factors. The *educational stressors hypothesis* contends that increased rates of stress are an unintended outcome of pronounced performance pressures placed on adolescents which emphasizes goal attainment.^15^ Swedish adolescents in the 9th grade can be characterized as a particularly stressful period due to the high-stakes nature of the transition from compulsory school to upper-secondary school. Individuals in the ninth grade must navigate varying demands in order to successfully transition into upper-secondary school in Sweden due to an increased focus on assessments and academic performance requirements under a ‘performance-based model’.^25^ Various studies have identified that such assessments are considered high-stakes and stress-inducing due to the subsequent costs this may have on their future academic prospects.^26–28^ As such, Swedish adolescents in the 9th grade are particularly vulnerable to experiencing stress.

Our findings further suggest that demands are associated with higher levels of stress among girls, which in turn is due to indirect effects of fear-of-failure. Recent evidence demonstrates that girls tend to fear living up to academic demands more than boys.^2^ Gendered norms regarding academic achievements suggest that girls hold greater academic aspirations than boys, and thus possess different interpretations of school-related demands and what is deemed to be ‘good enough’.^2^ Various findings further demonstrate that boys and girls report different attitudes to school and grades, as boys report investing less effort into school work, are more relaxed towards performance and are more satisfied with lower grades than are girls.^2^^,^^8^^,^^9^ As a result, girls likely increase academic effort, which causes achievement and expectations to be high, but simultaneously generates increased feelings of stress.^7^ Given that such norms may promote greater perceived school-related demands among girls, it is likely that girls experience increased fears-of-failure in failing to live up to such expectations. This is supported by recent findings which demonstrates that individuals who have high personal academic expectations possess increased levels of stress, as they fear living up to and meeting these expectations.^12^^,^^29^^,^^30^ Furthermore, embedded gender discrimination may also explain girls’ higher stress levels, as recent evidence suggests that girls are evaluated worse than boys across industries and occupations despite having identical academic qualifications.^31^ Therefore, girls are likely more reliant on possessing higher academic outcomes in order to compete, which in turn promotes higher levels of stress due to fears of failing to achieve the academic outcomes required to negate this gender inequality.

Explaining the inverse associations between both demands and fear-of-failure, and demands and negative affect among boys requires discussion. According to Martin (2013), experiences of fear-of-failure in the context of achievement likely promote various responses to how individuals perceive and cope with failure.^32^ Accordingly, two prominent dual-motives which explain responses to fear-of-failure are (i) *failure-avoidant* and (ii) *failure-accepting* behaviours.^33^ Failure-avoidant students are characterized by increased anxiousness motivated by fear-of-failure and are uncertain regarding their ability to avoid failure or achieve success.^32^^,^^33^ However, as a result of their motivation to avoid failure, failure-avoidant students tend to be studious, exert greater effort and possess higher levels of achievement, which ultimately results in increased levels of stress.^32^^,^^33^ Conversely, failure-accepting students are characterized by defensive pessimism, which involves setting lower or safer expectations, which likely reduces the threshold for performance expectations, in turn reducing fear-of-failure.^32^^,^^33^ Importantly, previous research has demonstrated that girls are more likely to demonstrate characteristics of failure-avoidant behaviours, while boys more likely fall into failure-accepting behaviours,^32^^,^^33^ which may explain the findings from our study that boys tended to experience reduced levels of fear-of-failure and negative affect when experiencing educational expectations. Further research is required to substantiate these findings.

Given the findings of this study, the implementation of evidence-based interventions which aid in mitigating the possible effects of educational expectations and fear-of-failure is required. Specifically, various psychological well-being programmes which aim to strengthen health and well-being factors have been trialed in various countries.^34^ One such programme is socio-emotional learning programmes, which aim to strengthen adolescents social and emotional competence, self-control, responsible decision-making and effective coping strategies.^34^ These programmes have demonstrated an overall medium effect on adolescents coping strategies and emotional well-being, with students also reporting involvement in such programmes improving their self-esteem, confidence, and led to an improved perception of the school environment.^34^ We additionally recommend removing or reducing eligibility requirements, which currently require students to achieve a minimum number of pass marks in grade 9 to access upper secondary school.^15^ Reducing such eligibility requirements would likely reduce students’ perceptions of demand, therefore reducing experiences of stress.

## Conclusions and limitations

This study identified a novel and distinct gender-specific difference in regard to the mediating role of fear-of-failure within the association between educational expectations and negative affect. The contributions of this study provide insights into the causes of gender differences in stress-related complaints. Previous research within an educational setting has consistently demonstrated the adverse effects associated with school-related demands and adolescent stress.^15^ Our study also has implications for the educational stressors hypothesis and educational policies in Sweden. Firstly, our study adds a previously uninvestigated dimension to the educational stressors hypothesis, by assessing how fear-of-failing interacts with educational expectations and negative affect. Secondly, our findings underscore the importance of assessing how current educational expectations in compulsory-school affect Swedish adolescents’ stress levels and the possible development of intervening strategies that may limit experiences of stress.

The results of our study should however be interpreted while taking its limitations into account. Firstly, our study utilized negative affect as a proxy for stress-related complaints. Though previous research has demonstrated that stress and negative affect are closely associated in regard to measurement consistency^17^, this may not be accurately measuring adolescent stress and may be distorting our findings. Furthermore, as negative affect is subjectively reported, adolescents’ perceptions may be masked by their developmental biological and psychological state at this age. Secondly, our study utilized only one measure to account for educational expectations, which may not account for the full complexity of expectations adolescents face, and how this interacts with experiences of adolescent stress. Thirdly, given that our study utilized cross-sectional data, it is difficult to determine the direction of causality, as fear-of-failure and or negative affect could affect educational expectations. Despite this limitation, our findings were nevertheless robust when adjusting for various confounders. However, longitudinal research investigating the direction of causality regarding educational expectations, fear-of-failure and negative affect is required. Lastly, the validity of the Swedish 2018 PISA results has received criticisms due to high exclusion criteria. Despite the PISA Adjudication Group concluding that Sweden’s exclusion rate resulted from an increase in immigration, and therefore not affecting the sample estimates of the Swedish sample, the possible effects on Sweden’s estimates should still be considered when assessing the findings of this study.

## Supplementary Material

ckad200_Supplementary_Data

## Data Availability

The data underlying this article are available in [Evidence Institute repository], at https://www.evidin.pl/en/data/. Key points Fear of failure partially mediates the relationship between educational expectations and negative affect, providing an increased understanding of the causes of adolescent stress. Girls are more vulnerable to experiences of stress when possessing higher educational expectations, which appears to increase due to increased levels of fear of failure in an educational setting. The findings from this study provide implications for public health policy and practice in better understanding potential causes of increased stress among adolescents in an educational setting.
