# Supplementary material for: Does fear-of-failure mediate the relationship between educational expectations and stress-related complaints among Swedish adolescents? A structural equation modelling approach
Source: Eur J Public Health. 2023 Nov 15;34(1):101–6. doi: 10.1093/eurpub/ckad200 (PMC10843945; doi:10.1093/eurpub/ckad200)
Supplement: ckad200_Supplementary_Data [file ckad200_supplementary_data.zip › ckad200_Supplementary_Data/ejph-2023-05-om-0223-File006.pdf]

1. Control variables

Reading achievement. We sought to control for the potential confounding effect of achievement on educational expectations utilising one indicator measuring students plausible value score in reading, which requires participating students to answer a variety of questions based on their comprehension of extended passages of text. This allows PISA to identify a range of proficiency levels related to reading comprehension.

Parental emotional support. We sought to control for the potential confounding effect parental emotional support may have on students educational expectations, utilising PISA parental emotional support index: "*To what extent do you agree with the following statements? My parents support my educational efforts and achievements; My parents support me when I am facing difficulties at school; My parents encourage me to be confident"*, with available response ranging from *(1) "strongly disagree"* to (2) "*strongly agree".* The statements were combined to create an index of parents' emotional support with low parental support being a score of 0, and high support being a score of 1.

Index of economic, social and cultural status. We sought to control for the potential confounding effects of individuals relative socio-economic position in society by utilising PISA's three way index of economic, social and cultural status, which is derived from parents highest level of educational attainment, parents highest occupational status, and number of home possession such as books in the home.

Immigtation: We sought to control for the potential confounding effect students immigration status may have on educational expectations by utilising one indicator assessing students immigration background, which is broken down by (1) native students who have at least one parent born in Sweden, (2) second generation students, who are born in Sweden but whoms parents migrated from another country, and (3) first generation students who were born outside of Sweden, and migrated to Sweden with their parents.

Social-media use: We sought to control for the potential confounding effect students relative use of social-media may effect their emotional well-being, by utilising one indicator which questions their social-media network use outside of school: *"Use of digital services outside of school: Participating in Social Networks (e.g. Facebook)"* with available responses ranging from (1) "*never or almost never"* to *(4) "once a week or more".*
