# Supplementary material for: Does fear-of-failure mediate the relationship between educational expectations and stress-related complaints among Swedish adolescents? A structural equation modelling approach
Source: Eur J Public Health. 2023 Nov 15;34(1):101–6. doi: 10.1093/eurpub/ckad200 (PMC10843945; doi:10.1093/eurpub/ckad200)
Supplement: ckad200_Supplementary_Data [file ckad200_supplementary_data.zip › ckad200_Supplementary_Data/ejph-2023-05-om-0223-File007.pdf]

Table S1: Model measuring the associations between fear of failure, educational expectations and negative affect and control variables

	Observed coefficient	Bootstrap std. err.	*z*	P>|z|	[95% conf. interval]	
FoF ← Edu-Exp	.044	.016	2.77	.006	.013	.074
FoF ← Emosups	-.081	.015	-5.35	.000	-.111	-.051
FoF ← ESCS	.036	.017	2.17	.030	.003	.068
FoF ← Immig	-.012	.016	-0.73	.467	-.045	-.021
FoF ← PV_Read	.074	.017	4.35	.000	.040	.107
FoF ← Social-M	.076	.015	4.76	.000	.044	.107
Neg-A ← FoF	.47	.014	34.79	.000	.447	.501
Neg-A ← Edu-Exp	.033	.015	2.19	.028	.003	.062
Neg-A ← Emosups	-.12	.014	-8.12	.000	-.147	-.089
Neg-A ← ESCS	-.015	.016	-0.91	.361	-.045	.017
Neg-A ← Immig	-.050	.016	-3.11	.002	-.081	-.018
Neg-A ← PV_Read	.068	.016	4.16	.000	.036	.099
Neg-A ← Social-M	.039	.015	2.56	.011	.009	.069

Note: FoF = Fear of Failure; Edu-Exp = Educational Expectations; Neg-A = Negative Affect; ESCS = Index of Economic, Social and Cultural Status; Emosups = Parental Emotional Support; Immig = Immigration Status; Social-M = Social Media Use; PV_Read = Plausible value in reading
